# Epithelioid Mesothelioma Cells Exhibit Increased Ferroptosis Sensitivity Compared to Non-Epithelioid Mesothelioma Cells

**DOI:** 10.3390/cancers17243983

**Published:** 2025-12-13

**Authors:** Tatsuhiro Sato, Ikue Hasegawa, Haruna Ikeda, Taichi Ohshiro, Lisa Kondo-Ida, Satomi Mukai, Satoshi Ohte, Tohru Maeda, Yoshitaka Sekido

**Affiliations:** 1 Division of Cancer Biology, Aichi Cancer Center Research Institute, Nagoya 464-8681, Japanr.kondo@aichi-cc.jp (L.K.-I.); smukai@aichi-cc.jp (S.M.); ysekido@aichi-cc.jp (Y.S.); 2 Department of Microbial Chemistry, Graduate School of Pharmaceutical Sciences, Kitasato University, Minato-ku 108-8641, Japan; ohshirot@pharm.kitasato-u.ac.jp (T.O.); ohtes@pharm.kitasato-u.ac.jp (S.O.); 3 Medicinal Research Laboratories, School of Pharmacy, Kitasato University, Minato-ku 108-8641, Japan; 4 Graduate School of Pharmaceutical Sciences, Kinjo Gakuin University, Nagoya 463-8521, Japan; phmaeda@kinjo-u.ac.jp; 5 College of Pharmacy, Kinjo Gakuin University, Nagoya 463-8521, Japan; 6 Department of Cancer Genetics, Nagoya University Graduate School of Medicine, Nagoya 466-8550, Japan

**Keywords:** mesothelioma, brefeldin A, RSL3, ferroptosis, epithelioid

## Abstract

**Background/Objectives**: Mesothelioma is a highly aggressive tumor with a poor prognosis that typically develops after a long latency period following asbestos exposure. Although immunotherapy combined with chemotherapy is increasingly used, the efficacy of standard treatments remains limited. This study aimed to explore ferroptosis induction as a potential therapeutic strategy for mesothelioma. **Methods**: We first screened microbial culture extracts collected from soil and marine environments to identify compounds with selective cytotoxicity against mesothelioma cells. Gene expression profiling was performed to investigate the mechanism of cell death induced by the identified compound. To assess intrinsic ferroptosis susceptibility, patient-derived mesothelioma cell lines and immortalized mesothelial cell lines were treated with RSL3, a GPX4 inhibitor. **Results**: Screening identified brefeldin A as a compound that selectively induces cell death in mesothelioma cells. Gene expression profiling revealed transcriptional changes consistent with ferroptosis induction. Treatment with RSL3 demonstrated marked variability in ferroptosis sensitivity across cell lines; the subgroup showing high sensitivity to RSL3 did not exhibit significant genetic alterations in *NF2* or *BAP1*, but contained a significantly higher proportion of epithelioid tumors in histological classification. **Conclusions**: Our findings highlight ferroptosis induction as a promising antitumor mechanism in mesothelioma, particularly in the epithelioid subtype. While GPX4 inhibitors such as RSL3 are effective in vitro, further studies are needed to overcome pharmacological limitations and define molecular determinants of ferroptosis susceptibility, which may inform future personalized therapeutic strategies.

## Simple Summary

Mesothelioma is a highly malignant tumor with a poor prognosis, yet treatment options remain limited. Current therapies, including chemotherapy and immunotherapy, show only modest efficacy, highlighting the urgent need for new strategies. This study revealed that brefelin A strongly inhibits the proliferation of mesothelioma cell lines compared to immortalized mesothelial cells by inducing ferroptosis. Furthermore, the GPX4 inhibitor RSL3 selectively induced cell death in a subset of mesothelioma cell lines, particularly those classified as the epithelioid subtype. These findings suggest that inducing ferroptosis could represent a promising antitumor mechanism in mesothelioma and may provide a basis for novel therapeutic approaches specifically targeting particular histological subtypes of mesothelioma.

## 1. Introduction

Mesothelioma is a rare and highly lethal tumor primarily associated with asbestos exposure [1,2,3]. Originating from mesothelial cells, approximately 70–80% of cases arise in the pleura, although the disease can also develop in the pericardium, peritoneum, and tunica vaginalis [4]. Genomic studies have revealed that mesothelioma is predominantly characterized by loss-of-function alterations in tumor suppressor genes, with mutations in *CDKN2A*, *BAP1*, and *NF2* being the most frequently observed [5,6]. *NF2* gene product, also known as Merlin, regulates the Hippo signaling pathway, and alterations in *LATS2*, another component of this pathway, have been detected in approximately 7–11% of cases [7]. According to the World Health Organization (WHO), mesothelioma is histologically classified into three major subtypes: epithelioid, biphasic, and sarcomatoid. Although previous studies have reported correlations between histological subtypes and molecular features, including *NF2* alterations [6,8] and the expression levels of *CLDN15* and *VIM* [5], the mechanisms underlying subtype specification remain largely unclear.

Most patients with mesothelioma are not considered suitable candidates for surgical intervention due to the extent of disease and the presence of comorbidities [9]. A recently reported MARS2 trial found no significant difference in overall survival between patients treated with extrapleural pneumonectomy plus chemotherapy and those treated with chemotherapy alone [10]. Subsequent expert analyses have pointed out several methodological limitations in this trial, complicating the interpretation of the results [11]. Nevertheless, current therapeutic strategies remain inadequate, and more effective therapeutic approaches are urgently needed.

For many years, the standard first-line treatment for mesothelioma has consisted of antifolate agents combined with platinum-based chemotherapy [12]. Recently, the emergence of immune checkpoint inhibitors has opened new avenues for therapeutic intervention. The CheckMate 743 trial demonstrated a significant improvement in overall survival with the combination of nivolumab and ipilimumab in patients with advanced pleural mesothelioma, compared to conventional chemotherapy [13]. In another trial, the DREAM study evaluated the combination of the anti–PD-L1 antibody durvalumab with standard chemotherapy (cisplatin or carboplatin plus pemetrexed), and also reported extended overall survival [14]. Despite these advances, the efficacy of current treatment options remains limited, underscoring the urgent need for innovative treatment strategies [15,16,17].

Several molecular targeted therapies have been explored for mesothelioma, particularly those aimed at tumors harboring *NF2* alterations. Focal adhesion kinase (FAK) inhibitors, for instance, were evaluated in clinical trials but did not demonstrate sufficient efficacy [18], with gene expression variability—such as differences in E-cadherin levels—being considered a contributing factor [19]. Similarly, TEAD inhibitors targeting the Hippo pathway are currently under clinical investigation. Although early-phase trials have demonstrated disease control in a subset of patients [20], the overall response rates remain modest. In addition, preclinical studies have reported transcriptional plasticity-driven resistance to TEAD inhibition in vitro, suggesting a potential limitation in therapeutic durability [21]. These observations suggest that therapeutic responses in mesothelioma may be influenced not only by genetic mutations but also by broader cellular characteristics.

Given the complexity of transcriptional variability in mesothelioma, testing compounds in genetically distinct cell lines may offer a practical way to explore context-dependent vulnerabilities. In this study, we conducted a screening of ethanol extract of microbial source to discover cytotoxic agents targeting mesothelioma, using three cell lines with different genetic alterations. Microorganisms produce unique secondary metabolites through evolutionary processes [22], and some of these compounds may exert biological effects via mechanisms distinct from those of known agents [23]. Microbial sources are particularly advantageous for screening, as they contain small molecules and can be applied directly without complex preprocessing. With this rationale, we report the identification of known compound brefeldin A [24] as a selective cytotoxic compound, together with ferroptosis-related vulnerabilities in a mesothelioma subgroup.

## 2. Materials and Methods

### 2.1. Cell Culture and Reagents

HOMC, ACC-MESO, and Y-MESO cell lines were established in our laboratory and are available from RIKEN BioResource Center, as described previously [21]. MSTO-211H, NCI series, and MeT-5A cell lines were purchased from the American Type Culture Collection. All cells were grown in RPMI-1640 medium (FUJIFILM Wako Pure Chemical, Osaka, Japan, catalog no. 189-02025) supplemented with 10% (*v*/*v*) fetal bovine serum (Thermo Fisher Scientific, Waltham, MA, USA, catalog no. 10270-106) and 1 × Penicillin-Streptomycin Solution (FUJIFILM Wako Pure Chemical, catalog no. 168-23191) at 37 °C in a humidified incubator with 5% CO_2_. Information regarding *NF2*, *BAP1*, and *LATS2* alterations, as well as histological classification of the cell lines, is summarized in Table A1. All cell lines were confirmed Mycoplasma-free using the MycoBlue Mycoplasma Detector Kit (Vazyme, Nanjing, China, catalog no. D101-02) and authenticated by short-tandem repeat analysis using the PowerPlex 16 HS System (Promega, Madison, WI, USA, catalog no. DC2101). Brefeldin A (BFA) was purchased from Cell Signaling Technology (catalog no. 9972). RSL3 (catalog no. S8155) and Ferrostatin-1 (catalog no. S7243) were purchased from Selleck Chemicals (Houston, TX, USA).

### 2.2. Cell Survival and Death Assays

Cell survival and cell death were assessed using Cell Counting Kit-8 and Cytotoxicity LDH Assay Kit-WST (Dojindo, Kumamoto, Japan, catalog no. CK04 and CK12, respectively), according to the manufacturer’s instructions. Phase confluence was quantified using the IncuCyte S3 imaging system (Sartorius). Cells were seeded into 96-well plates and imaged every 6 h using the integrated phase-contrast imaging module. Confluence values were automatically calculated using the IncuCyte analysis software (version 2021C). Dose–response curves for RSL3 treatment were plotted using nonlinear regression fitting to a sigmoidal model in GraphPad Prism 9 (GraphPad Software), from which IC_50_ values were calculated.

### 2.3. Preparation of Microbial Extracts

The microbes used for screening were cultured on multiple media. The specific media used and the detailed culture conditions for each isolate are provided in the Supplementary Materials (Table S1). The microbes were then extracted with 70% ethanol. After removing debris from the extracts, they were concentrated under reduced pressure to remove ethanol completely. These were redissolved in DMSO before use in assays.

### 2.4. Screening of Ethanol Extract of Microbial Source

Screening of ethanol extracts of microbial sources was performed using three mesothelioma cell lines (Y-MESO-27, NCI-H2052, and NCI-H2452) and one mesothelial cell line (MeT-5A), seeded in 96-well plates. On the day following seeding, ethanol extracts (*n* = 1188) were added at a final concentration of 1:100 (*v*/*v*). After 72 h of incubation, cell viability was assessed using the Cell Counting Kit-8. Extracts that induced ≥90% growth inhibition in at least one mesothelioma cell line were considered hits. A total of 144 extracts met this criterion and were subjected to secondary screening. For this, each extract was serially diluted in seven steps (1:100 to 1:100,000) and tested under the same conditions to evaluate dose-dependent effects.

### 2.5. Identification of Brefeldin A from Fungal Strain BF-0398

From this screening, fungal strain BF-0398, which was isolated from the soil sample collected at Tomamu, Hokkaido, Japan, was selected. To identify an active compound, ethanol extract of BF-0398 was fractionated by Sep-PAK C18 plus cartliage (Waters, Milford, MA, USA, catalog no. WAT020515). The active compound was eluted in the 30% CH_3_CN fraction and identified by in-house HPLC-photodiodo array (PDA) library as the known brefeldin A [24].

### 2.6. Microarray Analysis

Total RNA was extracted using the RNeasy Mini Kit (QIAGEN, Hilden, Germany) according to the manufacturer’s instructions. The gene expression profiles were analyzed with Aproscience Inc. (Toronto, ON, Canada) using 8 × 60 K v3 microarrays (Agilent Technologies, Santa Clara, CA, USA). Microarray data were deposited in the Gene Expression Omnibus (GEO) under the accession number GSE312181.

### 2.7. qRT-PCR

Quantitative RT-PCR analyses were performed as previously described [25]. qRT-PCR was performed in triplicate using the QuantStudio 3 system (Applied Biosystems, Foster City, CA, USA). Relative gene expression levels were standardized using ACTB and analyzed using the Pfaffl method [26]. The primer sequences for qRT-PCR were as follows: ACSL4 (forward: 5′-GCTATCTCCTCAGACACACCGA-3′, reverse: 5′-AGGTGCTCCAACTCTGCCAGTA-3′); SLC7A11 (forward: 5′-TCCTGCTTTGGCTCCATGAACG-3′, reverse: 5′-AGAGGAGTGTGCTTGCGGACAT-3′); GPX4 (forward: 5′-ACAAGAACGGCTGCGTGGTGAA-3′, reverse: 5′-GCCACACACTTGTGGAGCTAGA-3′).

### 2.8. Detection of Lipid Peroxide

Lipid peroxide was stained by Liperfluo (Dojindo, catalog no. L248) according to the manufacturer’s protocol. Cells seeded on 96 well plates were treated with 1 μM Liperfluo for 30 min at 37 °C, 5% CO_2_ and the fluorescence intensity of the cells was measured every 2 h for 72 h using the IncuCyte S3 imaging system.

### 2.9. Statistical Analysis

Statistical analyses were performed using Microsoft Excel and GraphPad Prism 9 (GraphPad Software). Specific statistical tests used for each experiment are described in the corresponding figure legends. *p* values < 0.05 were considered statistically significant. All experiments were performed independently at least three times.

## 3. Results

### 3.1. Identification of Brefeldin A as an Inhibitor of Mesothelioma Cell Proliferation

**Figure 1:**
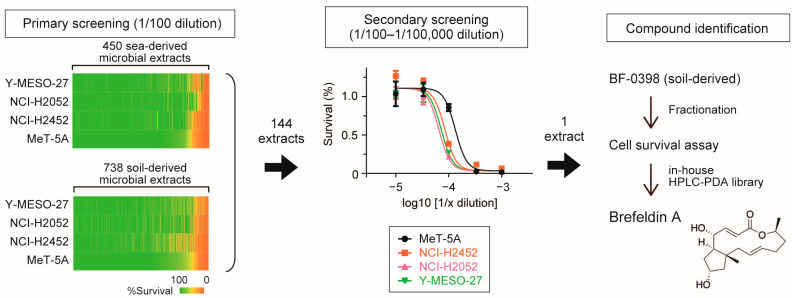
Identification of compounds inhibiting mesothelioma cell proliferation from microbial culture extracts. Ethanol extracts from 450 marine-derived and 738 soil-derived microorganisms were added at a 1/100 dilution to four cell lines. These cell survival was assessed after three days of culture and the relative survival rates are shown in heatmaps (**left panel**). Selected extracts were then subjected to serial dilution assays (1/100 to 1/100,000), with a representative dose–response curve for BF-0398 shown in the (**middle panel**). BF-0398 was subsequently fractionated, and the active compound was identified as brefeldin A based on the match of its retention time and PDA spectrum to our in-house HPLC–PDA library (**right panel**).

To explore novel cytotoxic agents against mesothelioma, we screened microbial culture extracts collected from soil and marine environments, focusing on their ability to suppress cell proliferation (Figure 1). Three mesothelioma cell lines were used in this assay: NCI-H2052 cells harboring *NF2* mutations, Y-MESO-27 cells with *LATS1/2* alterations, and NCI-H2452 cells carrying *BAP1* mutations. As a control, immortalized mesothelial cells (MeT-5A) were included. Among the screened samples, 144 culture broths that inhibited the proliferation of one or more mesothelioma cell lines were selected for further analysis using serial dilution. Notably, ethanol extract of fungal strain BF-0398 suppressed the growth of all three mesothelioma cell lines more effectively than that of MeT-5A cells, which exhibited reduced sensitivity across the tested dilution range. We therefore purified the active compound from this strain and identified it as brefeldin A (BFA), a macrolide antibiotic, based on its match to our in-house HPLC–PDA library.

### 3.2. Low-Dose Brefeldin A Induces Cell Death in Mesothelioma Cells

**Figure 2:**
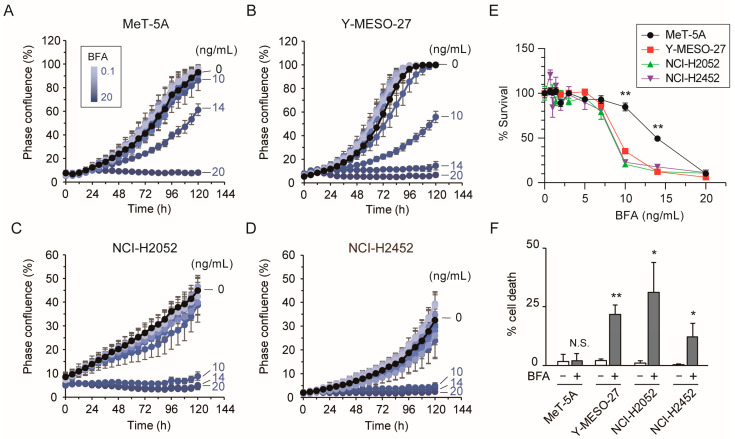
Low-dose BFA induces cell death in mesothelioma cells, but not in mesothelial cells. (**A**–**D**) Immortalized mesothelial MeT-5A cells (**A**) and three mesothelioma cell lines (**B**–**D**) were cultured with 0–20 ng/mL brefeldin A, and cell proliferation was monitored every 6 h for up to 120 h as cell confluence using the IncuCyte S3 live-cell imaging system. Data represent the mean ± SD of at least three independent experiments. (**E**) Cell viability after 96 h of culture with 0–20 ng/mL brefeldin A was assessed using the Cell Counting Kit-8. Data represent the mean ± standard deviation (S.D.) from three independent experiments. (**F**) The proportion of dead cells after 72 h of culture with 0 ng/mL (−) or 10 ng/mL (+) BFA was measured using the Cytotoxicity Assay Kit. Statistical significance between the two conditions was evaluated for each cell line using Student’s *t*-test, based on results from three independent experiments. N.S., not significant; *, *p* < 0.05; **, *p* < 0.01.

We next examined the time-dependent effects of BFA on cell proliferation using the commercially obtained compound. Treatment of MeT-5A cells with 10 ng/mL (approximately 36 nM) or lower concentrations of BFA showed no impact on cell growth for up to 120 h (Figure 2A). In contrast, 10 ng/mL BFA reduced the proliferation of Y-MESO-27 cells to approximately 40% by 96 h (Figure 2B), and almost completely inhibited the growth of NCI-H2052 and NCI-H2452 cells (Figure 2C,D). Cell viability assays revealed a significant reduction in survival of all three mesothelioma cell lines at concentrations ranging from 10 to 14 ng/mL BFA (Figure 2E), with greater sensitivity observed compared to MeT-5A cells. Consistent with its antiproliferative effects, 10 ng/mL BFA also induced cell death in the mesothelioma cell lines but not in MeT-5A cells (Figure 2F). These results indicate that BFA exerts a more potent and selective cytotoxic effect on mesothelioma cells than on mesothelial cells.

### 3.3. Low-Dose Brefeldin A Selectively Triggers Stress-Related Transcriptional Responses in Mesothelioma Cells

**Figure 3:**
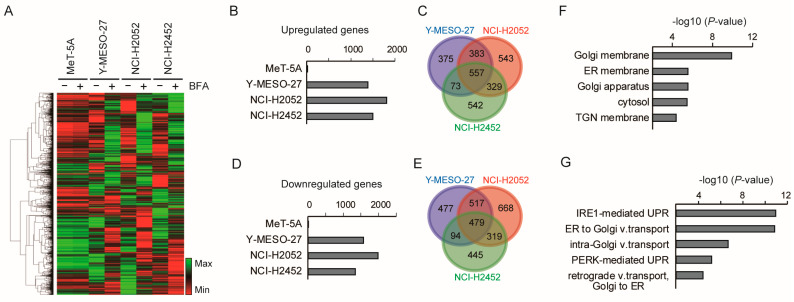
Microarray analysis identifies gene expression changes specific to mesothelioma cells in response to low-dose BFA. (**A**) Microarray analysis was performed using RNA extracted from cells cultured for 48 h with 0 ng/mL (−) or 10 ng/mL (+) brefeldin A. Genes showing more than twofold changes in expression upon BFA treatment are visualized in a heatmap. (**B**–**E**) From the dataset in (**A**), the number of upregulated genes in each cell line (**B**) and their overlap among the three mesothelioma cell lines (**C**), as well as the number of downregulated genes (**D**) and their overlap (**E**), are shown. Bar graphs are used in (**B**,**D**), and Venn diagrams in (**C**,**E**). (**F**,**G**) Gene Ontology (GO) enrichment analysis was performed using DAVID on the 557 genes commonly upregulated in the three mesothelioma cell lines shown in (**C**). The top five enriched terms are presented as bar graphs for cellular component (CC; (**F**)) and biological process (BP; (**G**)).

To further characterize the cellular response to BFA, we performed gene expression profiling using microarray analysis (Figure 3A). Notably, 10 ng/mL BFA induced substantial transcriptional changes in all three mesothelioma cell lines, whereas MeT-5A cells showed minimal responses (Figure 3B–E). Among the mesothelioma lines, 557 genes were commonly upregulated and 479 downregulated, suggesting a shared transcriptional program triggered by BFA in mesothelioma cells.

BFA is known to inhibit anterograde transport from the endoplasmic reticulum (ER) to the Golgi apparatus by interfering with ARF1-mediated vesicle formation [27,28]. To assess whether low-dose BFA is sufficient to elicit this effect in mesothelioma cells, we performed GO enrichment analysis on the commonly upregulated genes. The results revealed significant enrichment of genes localized to the ER and Golgi (Figure 3F), as well as genes involved in the unfolded protein response (UPR) and vesicular transport (Figure 3G). These findings demonstrate that low-dose BFA elicits stress-related transcriptional responses selectively in mesothelioma cells, highlighting a distinct cellular vulnerability not observed in immortalized mesothelial cells.

### 3.4. BFA Induces Ferroptosis in Mesothelioma Cells

**Figure 4:**
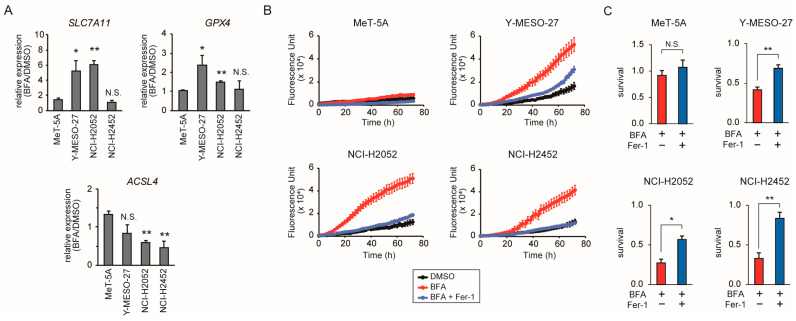
Low-dose BFA induces ferroptosis in mesothelioma cells. (**A**) Expression of *SLC7A11*, *GPX4*, and *ACSL4* in four cell lines after 72 h treatment with low-dose BFA (10 ng/mL) or DMSO, analyzed by quantitative RT-PCR. Relative expression levels (BFA/DMSO) are presented as a bar graph. Data represent mean ± SD of three independent experiments. (**B**) Cells were treated with low-dose BFA or vehicle control (DMSO), and lipid peroxidation was monitored using Liperfluo fluorescence quantified by the IncuCyte S3. Fluorescence intensity was measured every 2 h for 72 h. Data represent the mean ± SE of at least three independent experiments. (**C**) Cell survival after 72 h treatment with low-dose BFA was measured using Cell Counting Kit-8 in the presence or absence of the ferroptosis inhibitor Ferrostatin-1 (Fer-1, 10 μM). Data represent mean ± SD of three independent experiments. Statistical significance was determined by Student’s *t*-test. N.S., not significant; *, *p* < 0.05; **, *p* < 0.01.

As ER stress and UPR are known to influence various cell death pathways, including ferroptosis [29,30,31], we next investigated whether low-dose BFA affects the expression of ferroptosis-related genes in mesothelioma cells. Differential expression analysis based on microarray data revealed alterations in genes associated with ferroptosis. Quantitative RT-PCR analysis confirmed significant changes in the expression of key ferroptosis-related genes in mesothelioma cells cultured with low-dose BFA (Figure 4A). These included glutathione peroxidase 4 (GPX4), responsible for detoxifying lipid peroxides; SLC7A11, a cystine transporter essential for glutathione synthesis; and acyl-CoA synthetase long-chain family member 4 (ACSL4), which facilitates the incorporation of polyunsaturated fatty acids into membrane phospholipids. Expression changes were consistently observed in two of the three mesothelioma cell lines, indicating a reproducible transcriptional response to low-dose BFA.

These gene expression changes prompted us to investigate whether low-dose BFA functionally promotes ferroptosis in mesothelioma cells. Ferroptosis is a form of regulated cell death triggered by the peroxidation of polyunsaturated fatty acids (PUFAs) [32,33,34]. To assess lipid peroxidation directly, cells treated with low-dose BFA or vehicle control (DMSO) were stained with Liperfluo, a fluorescent probe that selectively detects lipid peroxides. All three mesothelioma cell lines exhibited strong fluorescence following BFA treatment, whereas MeT-5A cells, serving as a non-mesothelioma control, showed no increase in signal. Time-course analysis revealed progressive fluorescence accumulation in mesothelioma cells (Figure 4B), indicating that the signal reflected lipid peroxide buildup rather than nonspecific staining. Importantly, co-treatment with BFA and the ferroptosis inhibitor Ferrostatin-1 significantly suppressed cell death in all three mesothelioma cell lines (Figure 4C), demonstrating that ferroptosis contributes substantially to BFA-induced cytotoxicity.

### 3.5. Epithelioid Mesothelioma Cell Lines Exhibit Intrinsic Sensitivity to the Ferroptosis Inducer RSL3

**Figure 5:**
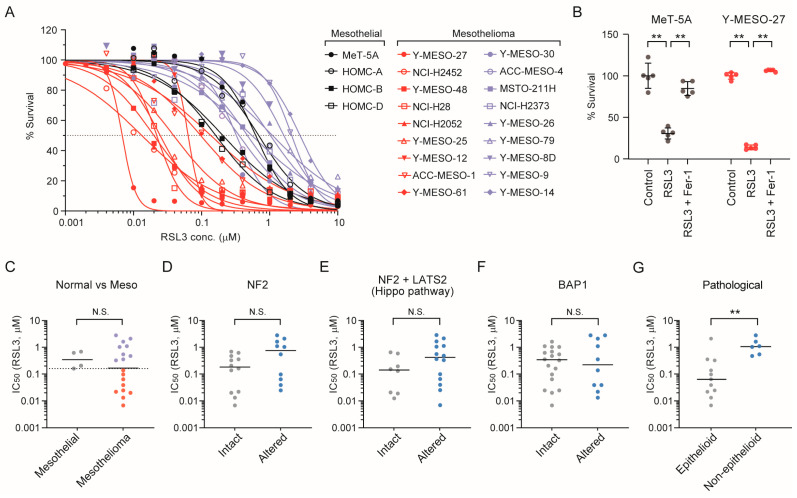
Ferroptosis sensitivity across mesothelioma cell lines and its association with genetic and histological features. (**A**) Dose–response curves of RSL3 in mesothelioma and normal mesothelial cell lines. Cell survival was measured after 96 h treatment and plotted against RSL3 concentration (μM). Data represent mean ± standard deviation (S.D.) from at least three independent experiments. The dashed line indicates 50% survival. Cell lines are color-coded for visual clarity; IC_50_-based classification is presented in Figure 5C. (**B**) Rescue of RSL3-induced cytotoxicity by Ferrostatin-1 (Fer-1) co-treatment in MeT-5A and Y-MESO-27 cells. Cell survival was assessed after 96 h. Data from five independent experiments are shown as individual dots, with mean ±SD indicated by overlaid lines. Statistical significance was determined by Student’s *t*-test. (**C**) IC_50_ values of RSL3 in mesothelial and mesothelioma cell lines were plotted. The dashed line indicates the lowest IC50 value among the four mesothelial cell lines. Red and purple indicate sensitive and resistant cell lines, respectively. (**D**–**F**) RSL3 IC_50_ values were compared between intact and altered groups for *NF2* (**D**), *NF2* and/or *LATS2* (**E**), and *BAP1* (**F**). Mesothelial cell lines were alla included in the intact groups. (**G**) RSL3 IC_50_ values were compared between mesothelioma cell lines of epithelioid versus non-epithelioid origin. Y-MESO-48 was excluded from this analysis due to unknown histological origin. Statistical significance was determined by Mann–Whitney U-test. N.S., not significant; **, *p* < 0.01.

Building on the functional evidence suggesting that low-dose BFA induces ferroptosis, we next sought to assess the intrinsic ferroptosis sensitivity of mesothelioma cells using RSL3, a direct GPX4 inhibitor. To this end, 18 patient-derived mesothelioma cell lines and 4 immortalized mesothelial cell lines were treated with various concentrations of RSL3 (Figure 5A). Ferroptosis induction was confirmed by the rescue of cell viability in MeT-5A and Y-MESO-27 cells upon co-treatment with Ferrostatin-1 (Figure 5B). Compared to the mesothelial cell lines (black in the graph), the mesothelioma cell lines exhibited a broad range of responses to RSL3. The three mesothelioma cell lines used in earlier figures all exhibited lower IC_50_ values than any of the four mesothelial cell lines. This observation is consistent with the findings in Figure 4, where these mesothelioma cell lines showed a pronounced susceptibility to ferroptotic cell death upon low-dose BFA treatment.

Based on IC_50_ values calculated from the data in Figure 5A, mesothelioma cell lines were classified as “sensitive” or “resistant” depending on whether their IC_50_ values were lower than those of the mesothelial cell lines (Figure 5C). To identify molecular features associated with this sensitivity, we examined the mutational status of genes commonly altered in mesothelioma, including *NF2*, *LATS1/2*, and *BAP1*. However, no significant differences in IC_50_ distributions were observed between mutant and wild-type groups for any of these genes (Figure 5D–F). Ferroptosis sensitivity was further examined in relation to histological subtype. Mesothelioma cell lines of epithelioid origin exhibited consistently lower IC_50_ values compared to those of non-epithelioid origin (Figure 5G), highlighting a potential intrinsic vulnerability of epithelioid mesothelioma cells to ferroptosis induction.

## 4. Discussion

In this study, we investigated the ferroptosis sensitivity of mesothelioma cell lines using two distinct inducers: the microbial-derived compound BFA and the GPX4 inhibitor RSL3. BFA selectively induced cell death in mesothelioma cells at low concentrations, accompanied by gene expression changes and lipid peroxide accumulation consistent with ferroptosis induction. RSL3 treatment further revealed that epithelioid mesothelioma cell lines exhibit intrinsic vulnerability to ferroptosis, highlighting a potential subtype-specific therapeutic opportunity.

Previous studies have suggested that BFA-induced ER stress may contribute to ferroptosis [35], although the precise molecular mechanisms remain unclear. In our study, low-dose BFA partially activated UPR-related genes in mesothelioma cells, but the extent to which UPR contributes to ferroptosis induction—and whether it can be therapeutically targeted—requires further investigation. Unlike BFA, RSL3 directly inhibits GPX4, bypassing complex stress-response pathways, and thus serves as a useful tool for probing ferroptosis susceptibility. However, its translational potential remains uncertain and is discussed further below.

Our study did not directly analyze dynamic changes in lipid metabolism, reactive oxygen species (ROS), or iron homeostasis, which are critical regulators of ferroptosis. Future studies incorporating high-resolution lipidomic profiling, such as comprehensive lipidomic analysis, may enable the identification of specific lipid species responsible for ferroptosis and reveal structural features of oxidized lipids. Additionally, combining these approaches with advanced imaging techniques to visualize ROS generation and intracellular iron distribution in real time could provide deeper mechanistic insights into the execution of ferroptosis.

While previous reports have implicated *NF2* and *BAP1* mutations in modulating ferroptosis sensitivity [36,37], our data did not reveal significant differences in RSL3 IC_50_ values between intact and altered groups for either gene. This discrepancy may reflect context-dependent factors that influence ferroptosis susceptibility beyond individual genetic alterations. Notably, our results demonstrated that mesothelioma cell lines of epithelioid origin exhibited consistently higher sensitivity to ferroptosis induction. Epithelioid mesothelioma is known to express elevated levels of Claudin-15 and other epithelial markers, whereas non-epithelioid tumors tend to exhibit mesenchymal features such as Vimentin expression [5]. These lineage-specific molecular characteristics may affect membrane composition, lipid metabolism, or oxidative stress responses, thereby contributing to ferroptosis vulnerability in a histotype-dependent manner.

Although GPX4 inhibitors like RSL3 are valuable tools for probing ferroptosis susceptibility in vitro, several challenges limit their clinical applicability. Concerns regarding safety, off-target effects, and pharmacokinetic limitations remain major barriers to therapeutic use. Moreover, the lack of tumor-selective delivery systems and reliable biomarkers for ferroptosis susceptibility complicates their implementation in clinical settings. Some FDA-approved drugs, such as sulfasalazine and sorafenib, have been reported to induce ferroptosis via xCT inhibition, but their efficacy and selectivity are limited [38,39]. Considering recent findings that tumor microenvironment stress factors may influence ferroptosis sensitivity [40], future efforts should aim to define the molecular and histological features that confer ferroptosis susceptibility in mesothelioma cells.

Importantly, this study is based solely on in vitro analyses, which do not capture the complex tumor microenvironment. In vivo, EMT, extracellular matrix remodeling, matrix detachment, and stromal–immune interactions can markedly alter ferroptosis susceptibility [41,42,43]. Microenvironment-derived stresses have been broadly suggested to influence ferroptosis regulation [44], raising the possibility that non-epithelioid mesotheliomas may exhibit ferroptosis vulnerability in vivo despite their limited sensitivity in vitro. Together, these findings highlight the importance of integrating in vivo and microenvironmental contexts in future studies to fully evaluate the therapeutic potential of ferroptosis induction in mesothelioma.

## 5. Conclusions

In summary, our study demonstrated that mesothelioma cells are more susceptible to cell death induced by brefeldin A and the ferroptosis inducer RSL3 than immortalized mesothelial cells. Notably, epithelioid mesothelioma cell lines exhibited greater intrinsic vulnerability to ferroptosis compared to non-epithelioid subtypes. These findings highlight ferroptosis as a promising therapeutic target in mesothelioma and suggest that elucidating the molecular determinants of ferroptosis sensitivity—such as lipid composition and redox regulation—may pave the way for the development of more effective and personalized treatment strategies.

## Data Availability

The microarray data generated in this study have been submitted to the Gene Expression Omnibus (GEO) and will be publicly available upon completion of the registration process. The accession number will be provided upon publication. Other datasets supporting the findings of this study are available from the corresponding author upon reasonable request.

## Supplementary Materials

The following supporting information can be downloaded at: https://www.mdpi.com/article/10.3390/cancers17243983/s1, Table S1: Media compositions and culture conditions for microbial isolates used in the screening.

## Abbreviations

The following abbreviations are used in this manuscript:

ACSL4	Acyl-CoA synthetase long-chain family member 4
BAP1	BRCA1 associated protein 1
BFA	Brefeldin A
DMSO	Dimethyl sulfoxide
ER	Endoplasmic reticulum
GO	Gene ontology
GPX4	Glutathione peroxidase 4
IC_50_	Half maximal inhibitory concentration
NF2	Neurofibromin 2
PUFA	Polyunsaturated fatty acid
ROS	Reactive oxygen species
RSL3	RAS-selective lethal 3
SLC7A11	Solute carrier family 7 member 11
UPR	Unfolded protein response

## Appendix A

**Table A1:** Characteristics of cell lines used in this study.

Cell Line	Cell Type	NF2 Alteration	LATS2 Alteration	BAP1 Alteration	Histology
ACC-MESO-1	Mesothelioma	Y			Epithelioid
ACC-MESO-4	Mesothelioma		Y		Epithelioid
HOMC-A	Mesothelial				
HOMC-B	Mesothelial				
HOMC-D	Mesothelial				
MeT-5A	Mesothelial				
MSTO-211H	Mesothelioma		Y		Non-epithelioid
NCI-H2052	Mesothelioma	Y	Y		Epithelioid
NCI-H2373	Mesothelioma	Y			Non-epithelioid
NCI-H2452	Mesothelioma			Y	Epithelioid
NCI-H28	Mesothelioma			Y	Epithelioid
Y-MESO-8D	Mesothelioma	No exp.			Non-epithelioid
Y-MESO-14	Mesothelioma	Y	Y	Y	Non-epithelioid
Y-MESO-25	Mesothelioma	Y		Y	Epithelioid
Y-MESO-26	Mesothelioma	Y	Y		Non-epithelioid
Y-MESO-27	Mesothelioma		Y		Epithelioid
Y-MESO-30	Mesothelioma		Y		Epithelioid
Y-MESO-48	Mesothelioma				Unknown
Y-MESO-61	Mesothelioma			Y	Epithelioid
Y-MESO-79	Mesothelioma	Y		Y	Non-epithelioid

Y indicates the presence of genetic alteration. Blank cells indicate no alteration. “No exp.” denotes lack of NF2 protein expression, and was treated as NF2 alteration. One cell line with “Unknown” histology was excluded from histology-based RSL3 sensitivity analysis.
