# Supplementary material for: Epithelioid Mesothelioma Cells Exhibit Increased Ferroptosis Sensitivity Compared to Non-Epithelioid Mesothelioma Cells
Source: Cancers (Basel). 2025 Dec 13;17(24):3983. doi: 10.3390/cancers17243983 (PMC12731133; doi:10.3390/cancers17243983)
Supplement: Supplementary file 1 [file cancers-17-03983-s001.zip › cancers-4007978-supplementary.pdf]

Table S1. Media compositions and culture conditions for microbial isolates used in the screening.

This supplementary table indicates the medium composition and culture conditions used for culturing the microbes for screening.

Materials:

Agar was purchased from Shimizu Food (Shizuoka, Japan). Brown rice was purchased from Kakashi Beikoku (Saitama, Japan). $CoCl_2 \cdot 2H_2O$, $CuSO_4 \cdot 5H_2O$, $FeSO_4 \cdot 7H_2O$, $Fe_2(SO_4)_3 \cdot nH_2O$, galactose, glucose, KH_2PO_4, K_2HPO_4, $Mg_3(PO_4)_2 \cdot 8H_2O$, $MgCl_2 \cdot 4H_2O$, polypeptone, soluble starch, sucrose, $ZnSO_4 \cdot 7H_2O$ were purchased from FUJIFILM Wako Pure Chemical Corporation (Osaka, Japan). $CaCO_3$, glycerol, KBr, $MgSO_4 \cdot 7H_2O$ were purchased from Kanto Chemical (Tokyo, Japan). Ebios® was purchased from Asahi Group Food (Tokyo, Japan). Ehrlich meat extract was purchased from Pharmaceutical Industrial (Tokyo, Japan). Konbu tea was purchased Gyokuroen Food Industry (Tokyo, Japan). Malt extract and peptone was purchased from Life Technologies Corporation (Detroit, MI, USA). Potato dextrose broth (PDB), tryptone, and yeast extract were purchased from Becton Dickinson (Sparks, MD, USA). Sea water was purchased from Deep Ocean Suruga Bay (Shizuoka, Japan). Solulys was purchased from Oriental Yeast (Tokyo, Japan).

Actinomycete

Seed medium (M1)

1.0% soluble starch, 0.4% yeast extract, 0.2% peptone, 0.1% $CaCO_3$, and tap water, pH unadjusted.

Production medium

M1 (M1-, M1 + and M1k)

1.0% soluble starch, 0.4% yeast extract, 0.2% peptone, 0.1% $CaCO_3$, 0.004% $Fe_2(SO_4)_3 \cdot nH_2O$, 0.01% KBr with or without 3.0% konbu tea (M1k) in tap water (M1−) or in sea water (M1+), pH unadjusted.

Fungi

Seed medium (GP)

2.0% glucose, 0.2% yeast extract, 0.5% polypeptone, 0.1% KH_2PO_4, 0.05% $MgSO_4 \cdot 7H_2O$, 0.1% agar, and tap water, pH 6.0.

Production medium

F4 medium (F4)

2.0% sucrose, 1.0% glucose, 0.5% solulys, 0.5% Ehrlich meat extract, 0.1% KH_2PO_4, 0.3% $CaCO_3$, 0.05% $Mg_3(PO_4)_2 \cdot 8H_2O$, 0.1% agar, 1.0% trace metal solution (1 mg/mL of each $FeSO_4 \cdot 7H_2O$, $MgCl_2 \cdot 4H_2O$, $ZnSO_4 \cdot 7H_2O$, $CuSO_4 \cdot 5H_2O$, and $CoCl_2 \cdot 2H_2O$ in water), and tap water, pH 6.0.

F38 medium (F38)

3.0% sucrose, 3.0% soluble starch, 1.0% malt extract, 0.3% Ebios®, 0.5% KH_2PO_4, 0.05% $MgSO_4 \cdot 7H_2O$, and tap water, pH 6.0.

BMCF1 medium (BMCF1)

50 g brown rice, 5 ml of the following solution: 2.4% PDB, 0.5% $MgSO_4 \cdot 7H_2O$, 0.5% K_2HPO_4, and 0.5% $Mg_3(PO_4)_2 \cdot 8H_2O$ in tap water.

BMCF2 medium (BMCF2)

1.0% glucose, 2.0% sucrose, 0.3% yeast extract, 0.3% pharma media, 0.1% $MgSO_4 \cdot 7H_2O$, 0.2% KH_2PO_4, and 0.2% K_2HPO_4 in sea water, pH 6.0.

Genmai medium (Gen)

50 g brown rice, 25 ml of the following solution: 1.2% PDB, 1.0% malt extract, 1.0% glucose and 1.0% peptone in tap water.

PDB medium (PDB)

2.4% potato dextrose broth and 0.1% agar in tap water, pH 6.0.

DW and SW medium (DW and SW)

1.0% galactose, 2.0% glycerol, 1.0% glucose, 0.25% tryptone, 0.25% yeast extract, 0.05% KH_2PO_4, and 0.01% $FeSO_4 \cdot 7H_2O$ in distilled water (DW) or sea water (SW), pH 6.0.

Table The list of screening broth of actinomycetes or Fungi.

Actinomycetes		Culture medium		
		M1-	M1+	M1k
KM33	1~25	✓	✓	✓
KM34	1~25	✓	✓	✓
KM35	1~25	✓	✓	✓
KM36	1~25	✓	✓	✓
KM37	1~25	✓	✓	✓
KM38	1~25	✓	✓	✓

'✓" means that the screening broth was made by culture medium

Fungi			Culture medium					
	F4	F38	PDB	DW	SW	Gen	BMCF1	BMCF2
BF-0031~0039	✓	✓					✓	
BF-0090~0039	✓	✓					✓	
BF-0230~0249			✓	✓	✓			✓
BF-0260~0320			✓	✓	✓			✓
BF-0331~0358			✓	✓		✓		
BF-0359~0380	✓	✓				✓		
BF-0381~0458	✓	✓					✓	

'✓" means that the screening broth was made by culture medium
